# Need for optimized dosages in the design of comparative clinical trials of anti-malarial drugs

**DOI:** 10.1186/s12936-022-04170-1

**Published:** 2022-06-20

**Authors:** Sanjeev Krishna, Peter G. Kremsner

**Affiliations:** 1 Centre for Diagnostics and Antimicrobial Resistance, Institute for Infection and Immunity, St George’s University of London, London, UK; 2 Institut für Tropenmedizin, Universitätsklinikum Tübingen, Tübingen, Germany; 3 Centre de Recherches Médicales de Lambaréné, Lambaréné, Gabon

**Keywords:** Artemether–lumefantrine, Children, Clindamycin, Kenya, Malaria, Quinine

## Abstract

We read with interest the publication on malaria treatment by Obonyo et al. (Malaria J 21:30, 2022). This commentary questions the methodology, especially the chosen time points of treatment outcome measures.

A recent clinical trial conducted on Kenyan children by Obonyo et al. compared malaria treatment with quinine (10 mg/kg quinine sulphate: bd) given with clindamycin (10 mg/kg: bd) for six doses with artemether–lumefantrine (administered in weight adjusted rounded dosages) [1]. According to the authors, only 44% (n = 80) of children given quinine + clindamycin had an adequate clinical and parasitological response compared with 97% (n = 166) in the artemether-lumefantrine treatment group.

The design of comparative clinical trials for anti-malarials should be based on optimized dosage regimes, which unfortunately Obonyo’s is not. For example, in an earlier study in Gabon, Ramharter et al. have confirmed adequate responses when 3-day regimens of quinine (15 mg/kg: bd) are combined with clindamycin (7 mg/kg: bd) for children with malaria [2]. The higher quinine dosage is clearly essential, because earlier clearance of parasites (a bioassay of quinine efficacy as clindamycin has a much slower mode of action) was less common (54% (98/182) compared with artemether–lumefantrine (1%; 1/171) in Obinyo’s trial.

Even with higher quinine doses used together with clindamycin, there is a much longer parasite clearance time compared with an artesunate and clindamycin regimen (mean clearance times 46 h vs 29 h for the latter combination), although the final day 28 cure rate is very high and similar in both groups [2].

Short term regimes of quinine + clindamycin have been used in many clinical trials. Altogether, randomized clinical trials and clinical observations in Brazil and in Gabon, have shown between 88 and 100% final cure rates without counting for reinfections on day 28. Thus, the true cure rate may be higher when PCR genotyping would be used to discount reinfections. Up to half of patients had positive thick blood smears on day 3 in these studies without any effect on final cure rate and need of rescue treatment [2–7].

The mean parasite clearance times ranged between less than 2 days in African adults with mild malaria and low parasitaemia on admission [5], and 65 h in African children with severe malaria and very high parasitaemias on admission [6]. This clearly confirms that persistent parasitaemia at day 3 is not at all useful in these circumstances in assessing treatment success or failure.

Identifying non-artemisinin containing combination treatments for uncomplicated childhood malaria is an important objective, but it must be met by implementation of appropriate trial designs with correct dosing in each of the treatment arms in comparative trials. Failure to do so exposes children to the risk of under treatment and of serious misinterpretation of the value of particular regimens.

## Data Availability

The authors declare that all data supporting the findings of this study are available within the article.
